# On the Information Content of Coarse Data with Respect to the Particle Size Distribution of Complex Granular Media: Rationale Approach and Testing

**DOI:** 10.3390/e21060601

**Published:** 2019-06-17

**Authors:** Carlos García-Gutiérrez, Miguel Ángel Martín, Yakov Pachepsky

**Affiliations:** 1 Department of Applied Mathematics, Universidad Politécnica de Madrid, 28040 Madrid, Spain; 2 USDA-ARS Environmental Microbial and Food Safety Laboratory, Beltsville, MD 20705, USA

**Keywords:** information entropy, particle size distribution, selfsimilar measure, simulation

## Abstract

The particle size distribution (PSD) of complex granular media is seen as a mathematical measure supported in the interval of grain sizes. A physical property characterizing granular products used in the Andreasen and Andersen model of 1930 is re-interpreted in Information Entropy terms leading to a differential information equation as a conceptual approach for the PSD. Under this approach, measured data which give a coarse description of the distribution may be seen as initial conditions for the proposed equation. A solution of the equation agrees with a selfsimilar measure directly postulated as a PSD model by Martín and Taguas almost 80 years later, thus both models appear to be linked. A variant of this last model, together with detailed soil PSD data of 70 soils are used to study the information content of limited experimental data formed by triplets and its ability in the PSD reconstruction. Results indicate that the information contained in certain soil triplets is sufficient to rebuild the whole PSD: for each soil sample tested there is always at least a triplet that contains enough information to simulate the whole distribution.

## 1. Introduction

Granular media resulting from sedimentation and/or fragmentation processes are of great interest in different fields of science, technology and industry. The particle size distribution (PSD) is a main characteristic of these granular media since it has a crucial influence on their physical properties. These media are formed by an enormous amount of particles and, in fact, any particle size within the size interval might potentially be represented in a sample, so that, the PSD may be considered as a continuous distribution. In spite of this, experimental data on this distribution is usually very limited. In the case of soil, a paradigmatic natural granular media, the distribution information is commonly reduced to three classical size fractions, clay, silt and sand [1]. A first natural question arising at this point is if there is a theoretical framework supporting the supposed information value of so limited experimental data. While several mathematical distributions have been used as PSD models [2], the PSD reconstruction from this extremely poor information needs a rationale based in some driving idea different from empirical fitting procedures.

To address this challenge, this work is focused on characterizing the PSD by a specific property which could satisfy a simple equation (differential, difference or dynamical). An outstanding example of this kind of approach is the pioneering work [3] in which a differential equation is proposed as a semiempirical model for the cumulative mass-size distribution Q(x) of certain granular media with grain size below a given limit *x*.

$$\frac{dQ}{d\;(\log x)}=\alpha Q$$

The differential equation is formulated for granular materials whose grain distribution is arranged in the same statistical manner for both the smaller and for the greater sizes and conformed in such a way that adding a portion of greater grains, the resulting distribution is geometrically similar to the previous one; using the terminology of the authors, they have the same *granulography*.

Interestingly, behind this *old* model swarm features nowadays recognized in many complex dissipative systems. Indeed the formation processes of some granular media have certain aspects that are present in the dynamics of dissipative systems. Fragmentation of particles together with other coupled processes, suggest that the use of energy and its storage takes place in the form of “information” or disorder in the particle sizes. There are two constrains: the available energy for fragmentation is limited and also the energy needed to fragment a particle has a power law dependence of the size of the particle [4]. The maximum entropy principle [5] states that, under certain rules of optimality and randomness, the system thus would reach the maximum level of disorder conditional to the constrains imposed on the process. Entropy maximization methods have already been used to explain the power-scaling nature of size distributions caused by sudden breakage [6]. According to Prigogine [7], the balance of entropy production in dissipative systems should produce a characteristic organization level in a stationary state. In the context of this paper, this corresponds with a characteristic PSD heterogeneity. Notably the term *granulography* used in [3] may be interpreted in a very similar way. These features suggest the use certain elements of Information and Complex Systems Theories in the study of complex granular media, with the goal of establishing a rational basis under which one can evaluate and test the information content of a small number of wide ranges from the distribution.

The paper is organized as follows: in Section 2 a differential information equation is proposed as a conceptual approach for the PSD of complex granular media. Under this approach, experimental data may be seen as initial conditions of the above differential information equation. In Section 3 the use of detailed soil PSD data, together with methods based in the above mathematical approach are used to test the ability of limited experimental data to generate a full reconstruction of the PSD.

## 2. The Differential Information Equation for the PSD

Instead of the differential equation for the cumulative distribution proposed in [3], we present a rather different type of differential equation framed in a typical quantity used in the description of complex systems: the information entropy (IE).

In mathematical terms, the PSD of granular media may be seen as a mass particle-size distribution μ supported in the interval *I* of grain sizes.

Limited information on PSD is usually provided as a list of size ranges that cover *I*. Grains sorted according to their size thus appear distributed in a partition of size classes $P=\{I_{1},I_{2},\ldots ,I_{k}\}$ defined by those ranges on the list. If the corresponding mass fractions are $p_{1}=\mu \left(I_{1}\right),p_{2}=\mu \left(I_{2}\right),\ldots ,p_{k}=\mu \left(I_{k}\right)$, respectively, the IE of the partition *P* is defined by [8]

(1) $$H_{\mu }\left(P\right)=-\sum_{i=1}^{k}p_{i}\log p_{i},$$

provided $p_{i}\log p_{i}=0$ if $p_{i}=0$.

The number $H_{\mu }\left(P\right)$ is expressed in information units (bits) and its extreme values are logk, which corresponds to the most even case, when all the intervals have the same cumulative mass; and 0, which corresponds to the most uneven case, when the whole mass is concentrated in a single interval.

The number $H_{\mu }\left(P\right)$ can be interpreted as a measure of heterogeneity. In fact, in [9] it is shown that any measure of heterogeneity having the natural properties for this purpose, must be a multiple of $H_{\mu }\left(P\right)$. Both, the physical hypothesis and the differential equation in [3] have an implicit recognition of scale invariant features. Also the term *granulography* used there agrees with the concept of heterogeneity, which has a precise formulation in mathematical terms, as it was said above. Thus, instead of the differential equation proposed in [3] for the cumulative distribution, we propose a different type of differential equation involving the IE.

If we consider all the partitions $P=\{I_{1},I_{2},\ldots ,I_{k}\}$ of the size interval *I* that support the mass particle-size distribution μ, we define

(2) $$H_{\mu }\left(r\right)=\inf\left\{H_{\mu }\left(P\right):\;diam\;P\leq r\right\}$$

where r>0 and diamP is the diameter of *P*, this is, the length of the greater subinterval of *P*.

The use of IE allows to formulate a natural property of many multiparticular granular media similar to the master property proposed in [3]: after an arbitrary sieving at a characteristic size scale *r*, the amount of information received is related to that received at an “inmediately previous” sieving. This relation can be encapsulated in the following initial value problem

(3) $$\left\{\begin{array}{c} \frac{d\;H_{\mu (r)}}{d\;(\log r)}=D,\\ H_{\mu }\left(r_{0}\right)=H_{0} \end{array}\right.$$

where *D* is constant and $H_{\mu }\left(r_{0}\right)$ is the information received at an initial sieving of characteristic size $r_{0}$. This is the model we propose for the quantitative description of the PSD.

Although formally this differential equation resembles the proposed in [3], it involves different variables and has a complete different meaning. The physical hypothesis stated by [3], in these new terms, signify that when one travels through the scales using the logarithmic transformation, i.e., changes the size scale, the information content increases on the multiplicative scale. In particular, the equation implies that the information is conserved through the scales.

Under the theoretical point of view, for any partition $P=\{I_{1},I_{2},\ldots ,I_{k}\}$, the coarse information content of the corresponding empiric data

$$p_{1}=\mu \left(I_{1}\right),p_{2}=\mu \left(I_{2}\right),\ldots ,p_{k}=\mu \left(I_{k}\right),$$

may be used to provide the initial condition

$$H_{0}=-\sum_{i=1}^{k}p_{i}\log p_{i}.$$

A first issue is to find out if there is a solution of the Equation (3) corresponding to these initial conditions. Theoretical results from Fractal Geometry [10,11] assure that for each set of empiric data there exists a unique selfsimilar measure, which is a particular solution of (3) using those measured data as initial condition.

Initial data has an static information content that can be calculated with Shannon’s entropy. But the information potential of initial data is to suppose that this static information content is mantained, at least statistically, across the scales, which is exactly what the model (3) implies. Also, there is a scaling behaviour in every natural granular media.

Moreover, it turns out, that in the general case ($p_{1}=\mu \left(I_{1}\right)$, $p_{2}=\mu \left(I_{2}\right)$, …, $p_{k}=\mu \left(I_{k}\right)$) this measure agrees with the proposed as a model for soil mass size particle-size soil distribution in [12]. The latter now appears founded under this approach, and also gives the possibility of simulation. Furthermore, this result links the fractal PSD model proposed in [12] with the model given more than seventy five years earlier in [3].

Nevertheless, the multiplicative cascade associated to each set of experimental data is unique, so a second issue is to study for which initial conditions the corresponding solution μ better reconstructs the real PSD of a certain granular media. This study will take account of which experimental initial conditions (fraction contents) storage greater information content and are most useful to retrieve the actual PSD. There rest of this work is devoted to the study of this problem in the particular case of soil.

## 3. Materials and Methods

### 3.1. Data

**Figure 1:**
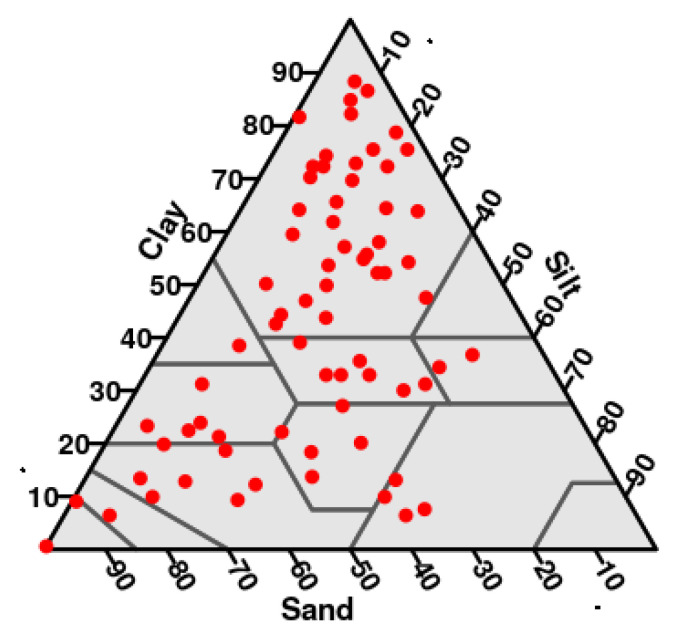
Representation of the 70 soil samples in the USDA textural triangle.

A total of 70 soil samples from the provinces of Jaen and Segovia in Spain were used. Samples were selected so that they covered the biggest possible part of the USDA textural triangle (Figure 1).

They belong to 10 different soil textural classes from the USDA textural classification [1], being the clay class the most represented, with 38 of the samples belonging to it. From a soil classification point of view, these soils belong to to 10 different soil classes, being Calcic Cambisol the most frequent. A complete description of these soils can be found in [13] and references within.

The particle size distribution of these samples was measured using the laser diffraction method [13] with the Longbench Mastersizer S (Malvern Instruments) with a He-Ne laser of 5 mW and a wavelength of 632.8 nm. This apparatus yields a set of data of the form

$${\left\{I_{i}=\left[\varphi_{i},\varphi_{i+1}\right],v_{i}\right\}}_{i=1}^{N},$$

where *N* is the total number of size intervals $I_{i}$, and $v_{i}$ is the percentage of total volume of particles whose sizes belong to the size interval $I_{i}$. Sizes are given in μm. Let *I* be the total size interval, i.e., $I=\cup_{i=1}^{N}I_{i}$. In this case I=[0.59−3473.45] Assuming a constant particle density the probability associated to each interval $I_{i}$ can be calculated as

$$p_{i}=\frac{v_{i}}{\sum_{i=1}^{N}v_{i}}.$$

The length of the size intervals, $I_{i}$, was not constant. The first interval is 0.12μm and the last one is 574.36μm. Nevertheless, when using a logarithmic scale, the interval sizes become even and the endpoints of the intervals verify that the quotient $\log\frac{\varphi_{i+1}}{\varphi_{i}}$ remains equal. Thus, we considered the following size intervals instead:

$${\left\{J_{i}=\left[\phi_{i},\phi_{i+1}\right]\right\}}_{i=1}^{N},\;\phi_{j}=\log_{10}\varphi_{j},\;j=1,2,\ldots ,N+1,$$

and *J* is the new total size interval in the logarithmic scale.

### 3.2. Simulation and Testing

Soil is a paradigmatic essentially complex granular system whose PSD is usually given in terms of the mass of only three size fractions, clay, silt and sand. These few data are are used as proxy for deriving many soil properties. Therefore it seemed natural to test sets of triplets of intervals along with their masses as initial conditions for the equation, simulate the whole distribution and compare it to a detailed description of the PSD. Indeed, different initial conditions lead to different PSD reconstructions. Testing differrent triplets would allow to find out if any of the them contains enough information to recover the whole PSD.

#### 3.2.1. Triplet Description

The hypothesis was tested using only three mass size intervals in the input partition.

By collapsing the detailed PSD description obtained through the experimental analysis different triplets of mass-size intervals were obtained to use as input data. With 48 available data intervals $\left\{J_{1},\ldots ,J_{48}\right\}$ along their respective masses $\left\{q_{1},\ldots ,q_{48}\right\}$ $\left(\sum q_{i}=1\right)$, the number of possible combinations of those intervals into a valid triplet $\left\{T_{1},T_{2},T_{3}\right\}$ was 1081. The mass of each input interval is the sum of all the masses from the data intervals that it comprises.

As an example, let $T_{1}=J_{1}\cup J_{2}$, $T_{2}=J_{3}$ and finally $T_{3}=\cup_{i=4}^{64}J_{i}$. Thus, the corresponding masses are $p_{1}=q_{1}+q_{2}$, $p_{2}=q_{3}$ and $p_{3}=\sum_{i=4}^{64}q_{i}$.

The geometric description of the triplets is the three intervals it comprises, i.e., $T_{1}=\left[a,b\right]$, $T_{2}=\left[b,c\right]$ and $T_{3}=\left[c,d\right]$. As *a* and *d* are the same for all triplets, the transformation

$$\alpha_{1}=\frac{b-a}{d-a},\;\alpha_{2}=\frac{c-a}{d-a},$$

allows for a succint representation of any triplet in the plane. The values of $\alpha_{1}$ ranged from $3.504\times 10^{-5}$ to 0.697. The values from $\alpha_{2}$ ranged from $7.375\times 10^{-5}$ to 0.835.

#### 3.2.2. Simulation Algorithm

To simulate the distribution using limited inputs, an iterated function system (IFS) was used [12]. This algorithm can simulate the mass within any interval A⊂J, being *I* the size interval provided by the laser diffraction analysis.

Once the initial interval is divided into the three size fractions that are going to be used as inputs: $T_{i}$, where $J=\cup_{i=1}^{3}T_{i}$, with their respective masses $p_{i}$, we shall calculate the linear transformations, $\xi_{i}$, i=1,2,3, that map *J* into $T_{i}$. Then the simulation algorithm is as follows:

1. take any $x_{0}\in I$ as a starting point,
2. choose randomly, with probability $p_{i}$, one of the three linear transformations $\xi_{i}$, i=1,2,3 and calculate the next point in the simulation $\xi_{i}\left(x_{0}\right)=x_{1}$,
3. repeat step (2), obtaining a sequence $\left\{x_{k}\right\}$ with $x_{k}=\xi_{i}\left(x_{k-1}\right)$ with probability $p_{i}$, chosen randomly, i=1,2,3.

This process defines a limit measure. The measure of any interval A⊂J, μ(A) can be calculated as

$$\mu \left(A\right)=\lim_{n\to \infty }\frac{m(n)}{n+1},$$

m(n) being the number of points of the orbit $\left\{x_{i}\right\}$ of *n* points that fall within the interval *A*.

The simulated distribution was statistically compared to the experimental one using a Kolmogorov-Smirnov (KS) test [14], and thus the distributions are statistically similar.

The convergence of the algorithm is fast. We performed simulations using increasing powers of ten points in the simulation. Only 0.34% of the triplets had a different KS test result when changing from $10^{5}$ to $10^{6}$ points in the simulation.

## 4. Results and Discussion

**Figure 2:**
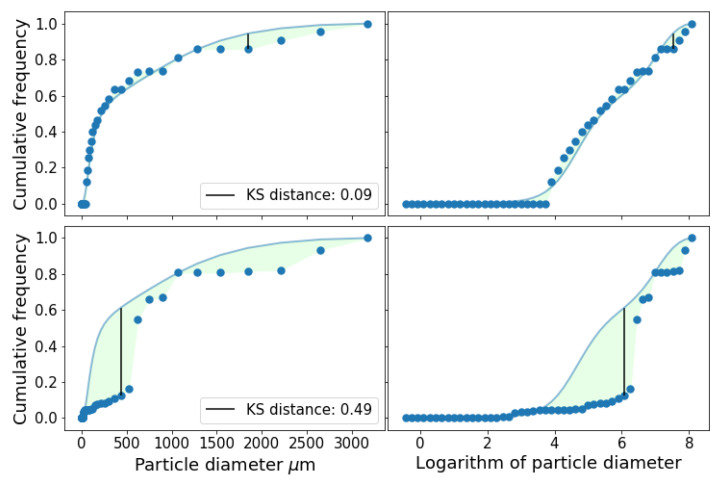
Actual (continuous line) and simulated (dots) PSD for soil 44. Top row shows the simulation using triplet $I_{1}=\left[0.59-1.46\right]$, $I_{2}=\left[1.46-1406.77\right]$ and $I_{3}=\left[1406.77-3473.45\right]$ μm. On the left the *x* scale is the particle diameter in μm, while on the right, for visualization purposes, it is on the logarithmic scale. For the bottom row, the input triplet used was $I_{1}=\left[0.59-37.84\right]$, $I_{2}=\left[37.84-1174.13\right]$ and $I_{3}=\left[1174.13-3473.45\right]$ μm. In both cases, the maximum allowed distance for the acceptance of the KS test at a 0.05 level was 0.28.

There was total of 1081 possible input triplets to simulate the whole PSD. At least 28 triplets (2.6%) passed the KS test for all soils in the database. On average 16.9% (∼182) of the available triplets passed the test for each soil. The number of triplets that passed the test was above 400 (>37%) for soils labeled 6 and 44. Example of simulation results is shown in Figure 2. Simulation with two different triplets is shown. The first one with input intervals $I_{1}=\left[0.59-1.46\right]$, $I_{2}=\left[1.46-1406.77\right]$ and $I_{3}=\left[1406.77-3473.45\right]$ in μm. The values of $\alpha_{1}$ and $\alpha_{2}$ for this triplet were 0.0003 and 0.4049.

The other triplet was $I_{1}=\left[0.59-37.84\right]$, $I_{2}=\left[37.84-1174.13\right]$ and $I_{3}=\left[1174.13-3473.45\right]$ μm, with $\alpha_{1}=0.0107$ and $\alpha_{2}=0.3379$.

**Figure 3:**
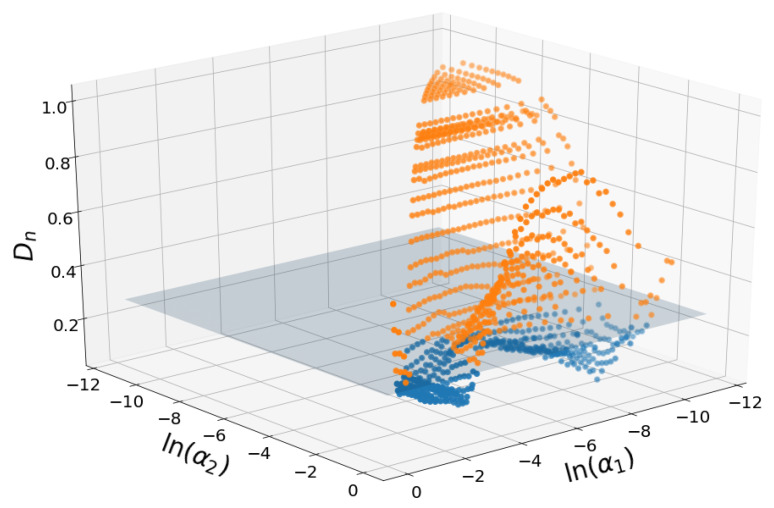
Representation of the KS distance, $D_{n}$, for all possible triplets, in the $\left(\alpha_{1},\alpha_{2}\right)$ plane, for soil 44. The values of $\alpha_{i}$ are in the log scale. The horizontal plane, at height, 0.28, is the limit value for $D_{n}$ for the acceptance region at the 0.05 level. Blue points, below the plane, are the ones that passed the test, while orange ones do not pass it. For this soil, 403 triplets (37.28%) pass the test.

Figure 3 shows the Kolmogorov-Smirnov statistics for all triplets represented in the $\left(\alpha_{1},\alpha_{2}\right)$ plane. The horizontal plane at the height of acceptance of the null hypothesis is shown.

The surface has butterfly-like shape with maximum values found at the extremes of the $\alpha_{2}$ values and intermediate $\alpha_{1}$ values. Either fine fraction (small $\alpha_{2}$) or coarse fraction (large $\alpha_{2}$) have to dominate to provide better modeling results.

**Figure 4:**
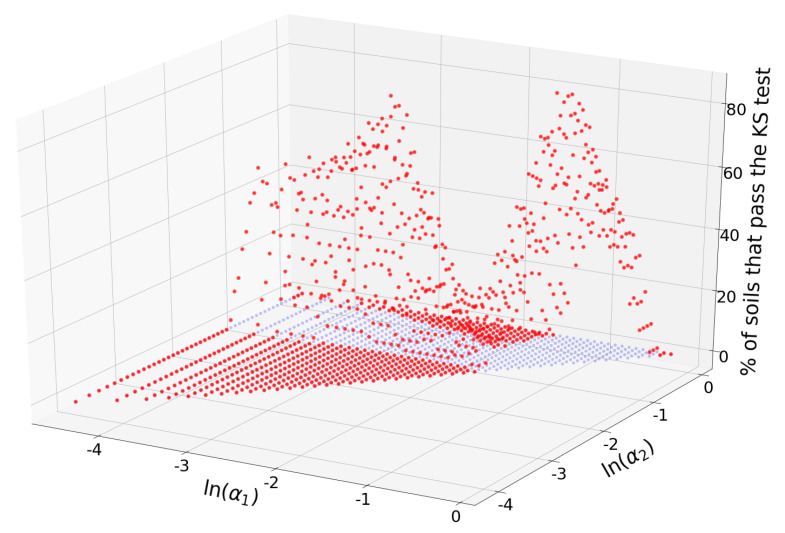
Red dots represent the percentage of samples that pass the KS test for a given triplet ($\alpha_{1}$,$\alpha_{2}$). For visualization purposes, the projection of the percentages on the plane have been added (blue dots).

Total of 536 (49.6%) of all the 1,081 available input triplets passed the KS test for at least one of the soils. The triplet that passed the KS test for most soils was $I_{1}=\left[0.59\;-192.61\right]$, $I_{2}=\left[192.61\;-\;979.85\right]$, $I_{3}=\left[979.85\;-\;3473.45\right]$ μm, with $\alpha_{1}=0.055$ and $\alpha_{2}=0.282$. The number of soils for which this triplet passed the KS test was 65 (93%). In terms of the USDA textural classification, this triplet consists of “clay + silt + fine sand”, “medium sand + coarse sand”, and “very coarse sand + gravel”. Total of 22 triplets passed the KS test for 55 soils (79%) or more. The percentage of soils for which each triplet $\left(\alpha_{1},\alpha_{2}\right)$ passes the KS test is shown in Figure 4.

**Figure 5:**
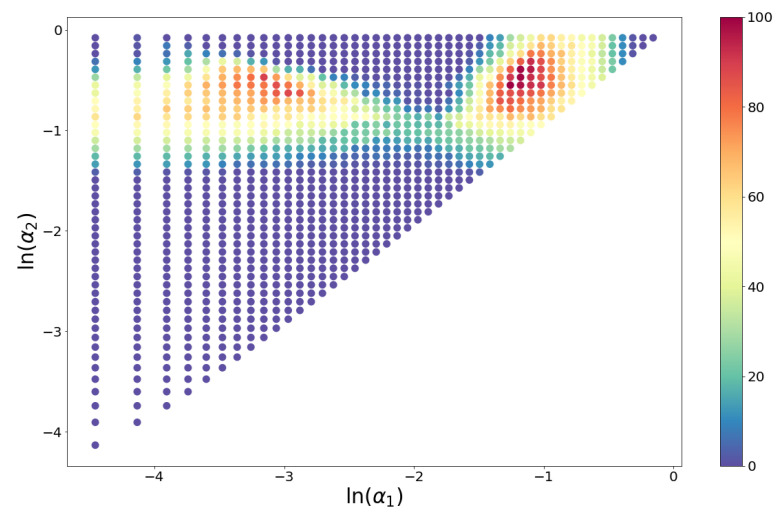
Heatmap for the percentage of samples that pass the KS test for a given triplet ($\alpha_{1}$,$\alpha_{2}$).

Triplets with maximum acceptance rates are concentrated in the neighborhood of the point $\left(\alpha_{1},\alpha_{2}\right)=\left(0.055,0.282\right)$, but there are other combinations of alpha values with relatively large acceptances. Figure 5 shows a heatmap of the acceptance percentage for the $\left(\alpha_{1},\alpha_{2}\right)$ plane.

Whether these points attractors have a general significance, are soil database specific or have relations with conditions of soil formation presents an interesting avenue for further research.

## 5. Conclusions

A new model is proposed for the PSD of granular systems. This model links two works that are separated almost 70 years in time. The link between both models is made by interpreting the driving idea of the first one by means of a differential information equation which leads to the second one. This model provides a theoretical based way to simulate the entire PSD using coarse textural data as initial conditions. This advantage allows to investigate which triplets (as coarse date) stores more information content, this measured by evaluating its potential in reconstructing the real PSD.

Any coarse description of the PSD can be used as an initial condition for the model. In particular, results indicate that the information contained in certain soil triplets is sufficient to reconstruct the whole PSD. For each soil tested there is always at least one triplet that contains enough information to simulate the whole distribution.

## Abbreviations

The following abbreviations are used in this manuscript:

PSD	Particle Size Distribution
IE	Information Entropy
KS	Kolmogorov-Smirnov
USDA	United States Department of Agriculture
